# Professor Parker’s Letter from Paris

**Published:** 1837

**Authors:** 


### Art. II.—Professor Parker.

Our last number announced the departure of this gentleman, early in the month of March, for Europe; for the double purpose of visiting the great hospitals of Paris and London, and of purchasing books, apparatus and preparations, for the Medical Department of the Cincinnati College. He will return before the commencement of the next session. It will be seen by the following extract from one of his letters, that he passed safely through the perils of the ocean, and was busily occupied on the important objects which led him to encounter them. D.

"PARIS, May 17, 1837.

"Since I have been here, I have been very much engaged among the hospitals, and on other matters of medical interest. I am, indeed, employed from half past six in the morning, till half past nine in the evening. The liberality of the French government towards institutions of Science, Literature and Charity, is unparalleled. Take the ROYAL INSTITUTE OF FRANCE as an example. A decree during the reign of terror, 1793, abolished all the academies that then existed, and has existed, from the time of Louis XIII; but on the 3d. Brumaire, 1795, the INSTITUTE was founded, and divided into three classes: 1st. Physical and Mathematical; 2d. Moral and Political; 3d. Literature and the Fine Arts. Bonaparte added a fourth class, viz: Ancient History and Literature. Louis XVIII. substituted the name of academy for class. In 1832, a fifth academy was created. So they now stand, 1st. *Academie Francaise;* 2d. *A. Royale des Inscriptiones et Belles Lettres;* 2d. *A. Royale des Sciences;* 4th. *A. Royale des Beaux Arts;* 5th. *A. des Sciences Morales et Politiques.* These academies, which together compose the ROYAL INSTITUTE, embrace 217 members, and each member is paid annually, by government, 1500 *francs.* The Academy of Sciences contains 63 members, and is the largest of the five. It meets every Monday, and all improvements, or attempts at improvement in science, are referred to it, and a report is made. Majiende is the president, which is regarded by the French as the highest scientific post of honor in the world. All the branches of the Institute are sustained by the government.

Again; to illustrate the liberality of this country, take the COLLEGES, of which there are 14 in Paris. The Royal College stands at the head. This has three departments: Theology, Law and Medicine. In the department of Medicine, there are 26 professors, and each receives from government from 2 to 8,000 *francs* a year: and the students have no expense to meet for instruction. There are seldom less than 5,000 medical students in the city. To be graduated, the young man must previously have received the degree of Bachelor of Arts, and must have studied four years. He is examined, *publicly*, at the end of each year. I have attended several of these examinations.

The professors vary much in their lecture room attractions. The amphitheatre will hold from fifteen to eighteen hundred. I have seen it at the lecture of Dumeril or Broussais, with only 50; while at Cruvilhier's, Andrals, Orfila's, or Magolin's, you will find the amphitheatre crowded to overflowing.

The law department is furnished by government with professors, and students attend without charge, and obtain diplomas as in medicine. Each young man, in order to graduate, must have previously taken the degree of Bachelor of Arts.

It is said, there are between 60 and 70,000 young men here, obtaining an education at the expense of the public, and by donations.

Again; I believe this country is surpassed by no other, and I doubt very much its being equalled by any other, in the extensive and liberal provision made for the accommodation of the wounded, sick, poor and helpless. Hospitals and hospices have been provided in a manner that reflects the highest credit upon the country. Both of these establishments are under the same direction, and based upon the same fund. The former are for the wounded and sick; the latter for incurables, the poor, &c. &c. Besides, there are *Bureaus* in different parts of the city, where prescriptions are made, for such as do not wish to enter those establishments; and, moreover, at each hospital, there is a room for the reception of out-door patients, where they are prescribed for. Biett is the great man here in chronic diseases of the skin. Every Monday he devotes from two to three hours to these patients; and I have seen 250 pass in review before him, on one of these days.

The *Hotel Dieu* has the greatest number of beds, about 1,500—others have about 100. The beds of all the hospitals together, amount to 5,337; those of the hospices to 11,740. These establishments cost the government, annually, 10,186,388 *francs*. The wards of these establishments are in very perfect order. Many of the bedsteads are of iron. They are furnished with good linen. Nor are the patients situated as in the time of Desault, when six or eight were placed in the same bed—the dead and dying together. In these establishments is employed the highest order of skill in the kingdom; and here we meet with every variety of disease. The hospital of St. Louis, where we find Alibert, Biett and Lugol, is one of much interest, as is also the Hospital Neckar, on account of Civiale's lithontriptic operations, which are carried on there. I have seen from three to six patients under his charge at one time; but I have not space to give you my opinion of his method.

I have procured, and am procuring books for our college daily; many of them the latest editions. I obtain them for one-third what we should be obliged to pay in our country. Some of the works, however, which our order embraces, are very expensive, especially Cruvilhier's great work with plates, and Alibert's on the maladies of the skin. The large dictionaries, also, are expensive.

I find instruments and chemical apparatus much cheaper in this country than in ours. I shall obtain a large assortment. The apparatus for injecting the absorbents must be got in London. I may not be able to obtain here *all* the morbid specimens we desire.

I have seen many interesting operations—have attended a course of lectures on diseases of the eye, and am attending other courses on Auscultation and Lithontripsy.

I have not seen our minister, Gen. Cass, as he is gone to Constantinople. I shall leave Paris for the south of France, Rome, and perhaps Naples, about the middle of June, and expect to be in England by the first of August. I hope every thing is going prosperously with our college."

